# Categorisation of continuous risk factors in epidemiological publications: a survey of current practice

**DOI:** 10.1186/1742-5573-7-9

**Published:** 2010-10-15

**Authors:** Elizabeth L Turner, Joanna E Dobson, Stuart J Pocock

**Affiliations:** 1 Department of Medical Statistics, London School of Hygiene & Tropical Medicine, London, UK

## Abstract

**Background:**

Reports of observational epidemiological studies often categorise (group) continuous risk factor (exposure) variables. However, there has been little systematic assessment of how categorisation is practiced or reported in the literature and no extended guidelines for the practice have been identified. Thus, we assessed the nature of such practice in the epidemiological literature. Two months (December 2007 and January 2008) of five epidemiological and five general medical journals were reviewed. All articles that examined the relationship between continuous risk factors and health outcomes were surveyed using a standard proforma, with the focus on the primary risk factor. Using the survey results we provide illustrative examples and, combined with ideas from the broader literature and from experience, we offer guidelines for good practice.

**Results:**

Of the 254 articles reviewed, 58 were included in our survey. Categorisation occurred in 50 (86%) of them. Of those, 42% also analysed the variable continuously and 24% considered alternative groupings. Most (78%) used 3 to 5 groups. No articles relied solely on dichotomisation, although it did feature prominently in 3 articles. The choice of group boundaries varied: 34% used quantiles, 18% equally spaced categories, 12% external criteria, 34% other approaches and 2% did not describe the approach used. Categorical risk estimates were most commonly (66%) presented as pairwise comparisons to a reference group, usually the highest or lowest (79%). Reporting of categorical analysis was mostly in tables; only 20% in figures.

**Conclusions:**

Categorical analyses of continuous risk factors are common. Accordingly, we provide recommendations for good practice. Key issues include pre-defining appropriate choice of groupings and analysis strategies, clear presentation of grouped findings in tables and figures, and drawing valid conclusions from categorical analyses, avoiding injudicious use of multiple alternative analyses.

## Background

A primary goal of observational epidemiology is to assess the strength, direction and shape of relationships between risk factors (exposures) and disease outcomes using appropriate statistical methods. Reports of such studies often categorise (group) continuous variables i.e. risk factors, health outcomes or confounders. In this article we focus on the categorisation of continuous risk factors.

There has been much methodological research into the practice of categorisation covering topics such as dichotomisation [1,2], efficiency loss and the effects of categorisation [3-9], reasons not to categorise [10,11] and flexible modelling methods to avoid categorisation [12,13]. A survey of reporting practices in the epidemiological literature [14] found categorisation to be common (84% of articles with a continuous risk factor used some form of categorisation). However, other than the limited information provided by this previous study, there is no documented evidence of how categorisation is carried out in published epidemiology and whether its planning, analysis and presentation are performed satisfactorily.

In this article we present an illustrative survey of recent epidemiological literature regarding such categorisation, including examples of both good and bad practice. We then present a series of recommendations for the practice of categorisation drawing from the lessons provided by the survey, from the broader literature and from experience. Such guidelines complement the STROBE guidelines [15] for the reporting of observational studies in epidemiology, in particular recommendation 11 which suggests that authors "*Explain how quantitative variables were handled in the analyses. If applicable, describe which groupings were chosen and why*".

## Methods

For five major epidemiological journals (American Journal of Epidemiology, Annals of Epidemiology, Epidemiology, International Journal of Epidemiology, Journal of Clinical Epidemiology) and five general medical journals (Annals of Internal Medicine, British Medical Journal, Journal of the American Medical Association, Lancet and New England Journal of Medicine) we reviewed two months' issues (December 2007 and January 2008), identifying all articles in observational epidemiology that examined in individuals the relationship between continuous risk factors and health outcomes. For the purpose of this survey, we defined a "continuous" risk factor as one which had at least 10 levels on an ordinal scale. If an article contained more than one main continuous risk factor, the first mentioned in the abstract was chosen. If more than one categorisation was performed, we focused on the most prominently featured (typically the first mentioned in the abstract). We considered only original research and excluded articles of randomised controlled trials, meta-analyses, studies where the analyses were not performed in individuals, agreement studies and studies where the main research question related to an effect modifier or interaction. Eligible articles had reporting of sufficient detail to ascertain how the continuous risk factor was analysed.

Two authors (ELT and JED) independently completed a proforma (piloted and agreed by SJP) for each eligible study using a standard form with pre-coded boxes and open text fields (Appendix 1 and Additional file 1). Any inconsistencies were reconciled by agreement where possible, or were resolved by the third author (SJP).

## Results

### Overall survey findings

**Figure 1:**
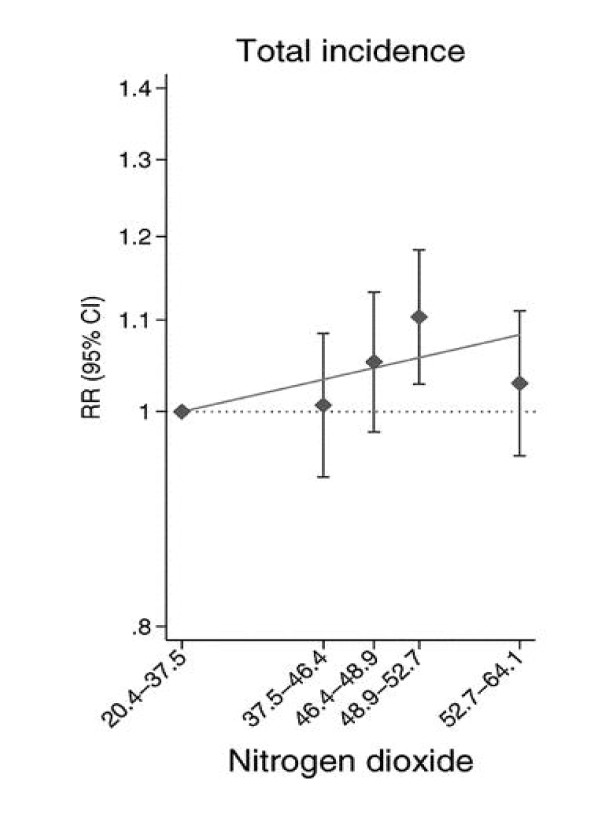
**Example from the survey: categorical results displayed as a figure**. Relative risk (& 95% CI) for coronary events by quintiles of NO_2_ (μg/m^3^) exposure [17]. Reference category is bottom fifth, trend line is fitted. More emphasis given to quantitative analysis. This image is reproduced with permission from *Epidemiology*.

Of the 254 reviewed articles from the 10 selected journals we identified 58 eligible articles published in December 2007 or January 2008 which studied one or more continuous risk factors (Table 1) (see Additional file 2 for a complete reference list). The five epidemiological journals contributed 47 eligible articles, whilst the five general medical journals contributed 11 eligible articles. The American Journal of Epidemiology contributed by far the largest number of articles (n = 31). The median number of subjects was 4,273 (inter-quartile range: 1,776 to 23,044; range: 212 to 1,487,223). Most (n = 31, 53%) articles presented results for cohort studies, 17 (29%) for cross-sectional studies and 10 (17%) for case-control studies. Outcome variables analysed were most commonly binary (n = 27, 47%), followed by time-to-event variables (n = 18, 31%) and continuous (n = 9, 16%). For the analysis of the main continuous risk factor, 8 (14%) articles presented a continuous analysis with no categorisation. For example, Vidula et al. [16] examined the relationship between biomarkers of inflammation and mortality using Cox proportional hazard modelling to obtain a hazard ratio per 1 unit increase in the biomarker. A total of 29 (50%) articles reported only an analysis of the categorised form of the continuous exposure whilst the remaining 21 (36%) articles presented both categorical and continuous analyses. For example, Rosenlund et al. [17] studied the relationship between estimated residential N0_2 _exposure and first coronary event using both categorical and continuous analyses (Figure 1). The former was used to evaluate any non-linearity of estimated effects (using quintiles). The authors' emphasis was placed on the results of the continuous analysis, with only these results presented in the abstract. Tsai et al. [18] studied the effects of obesity on absence from work using categorical analysis only.

**Table 1:** Main features of the 58 eligible articles with a continuous risk factor.

*Characteristic/Feature*	*Number of Articles n = 58*
	
Journal (number of articles reviewed^a^)	
American Journal of Epidemiology (48)	31 (53%)
Annals of Epidemiology (23)	9 (16%)
Epidemiology (16)	6 (10%)
Journal of the American Medical Association (26)	4 (7%)
New England Journal of Medicine (32)	3 (5%)
Annals of Internal Medicine (11)	2 (3%)
British Medical Journal (24)	1 (2%)
International Journal of Epidemiology (16)	1 (2%)
Lancet (49)	1 (2%)
Journal of Clinical Epidemiology (9)	0
	
Number of participants	
< 1,000	10 (17%)
1,000-5,000	23 (40%)
5,000-20,000	8 (14%)
20,000-100,000	11 (19%)
> 100,000	6 (10%)
	
Study Design	
Cohort	31 (53%)
Cross-sectional	17 (29%)
Case-control	10 (17%)
	
Type of outcome	
Binary	27 (47%)
Time to event	18 (31%)
Continuous	9 (16%)
Ordered categorical	2 (3%)
Unordered categorical	2 (3%)
	
Type of analysis for continuous exposure variable	
Categorically only	29 (50%)
Both continuously and categorically	21 (36%)
Continuously only	8 (14%)
	

Journal issues from December 2007 to January 2008 were reviewed.

^a ^All original research articles in each journal issue

### Characteristics of Categorisation

*Number of categories*. Of the 50 articles in which categorisation of the main continuous risk factor was used, most (76%) presented one choice of categories (Table 2). The remainder (24%) presented two categorisations (none presented more than two) of which we focus on that given greater prominence. Matsunga et al. [19] examined the relationship between ambient formaldehyde levels and allergic disorders such as atopic eczema. They used two forms of categorisation, the primary analysis with four groups and the secondary analysis with two groups. An extract of the results table is reproduced in Table 3. This example illustrates how interpretation based solely on hypothesis tests at a fixed significance level (not recommended), may lead to differing results from the alternative categorisations: in this case for a test at the 5% significance level the null of no effect would be rejected when the two group analysis was used (95% CI for estimated OR of 2.25: 1.01-5.01) but not for the four group analysis (p-value for trend = 0.08). Both results were described in the study abstract; the four-group analysis featured most prominently.

**Table 2:** Categorisation characteristics of the main continuous risk factor.

*Categorisation Characteristics*	*Number of Articles n = 50*
	
Number of categorisations used	
One	38 (76%)
Two	12 (24%)
Three or more	0
	
Number of categories^a^	
2	3 (6%)
3	9 (18%)
4	17 (34%)
5	13 (26%)
6	4 (8%)
7 to 10	3 (6%)
Unknown^b^	1 (2%)
	
Choice of category boundaries	
Quantiles	17 (34%)
Equally spaced intervals	9 (18%)
External criteria	6 (12%)
Other	17 (34%)
Unknown^b^	1 (2%)
	
"Zero/Never" category	10 (20%)
	
Presentation of categorical results	
Tables only	37 (74%)
Figures only	5 (10%)
Both Tables and Figures	5 (10%)
Neither	3 (6%)
	

For the sub-set of articles where categorisation was performed (n = 50 articles).

^a ^Of primary form of categorisation when more than one form was used

^b ^One article stated that categorisation was used but no results were presented

**Table 3:** Example of categorisation from the survey (1).

Formaldehyde levels (ppb)		Adjusted	
	Prevalence (%)	Odds Ratio (95% CI)	Odds Ratio (95% CI)
< 18	15/298 (5.0)	1.00	
18-27	15/299 (5.0)	1.03 (0.47-2.29)	1.00
28-46	17/301 (5.7)	1.11 (0.50-2.42)	
≥47	10/100 (10.0)	2.36 (0.92-6.09)	2.25 (1.01-5.01)
		P-value for trend = 0.08	

Prevalence of atopic eczema by formaldehyde levels [19].

Reason for inclusion as an example. Two alternative groupings: 4 groups (split at 30th, 60th and 90th percentile) and top 10% versus the rest. Quantitative analysis also presented but not reported in abstract. P-value for trend calculated using category medians.

Dichotomisation (two categories) featured most prominently in only three articles, two of these also used a second more detailed categorisation and the third also gave a continuous analysis. Catov et al. [20] examined the relationship between inflammation and pre-term birth by dichotomisation of inflammation with no alternative grouping presented (Table 4). Most studies (n = 39, 78%) used three, four or five categories. Seven articles (14%) used more than five categories. For example, in examining the relationship between birthweight and intellectual disability in 219,877 individuals, Leonard et al. [21] used seven categories of percentage of optimal birthweight (Table 5).

**Table 4:** Example of categorisation from the survey (2).

		34- < 37 weeks				< 34 weeks		
Inflammation	No.	Prevalence (%)	Adjusted Odds Ratio	95% CI		Prevalence (%)	Adjusted Odds Ratio	95% CI
No	279	20.8	1.0			8.6	1.0	
Yes	58	31.0	1.9	1.0,3.7		15.5	2.0	0.8,4.9

Inflammation (C-reactive protein≥8 μg/ml) before 21 weeks' gestation and risk of spontaneous pre-term birth by preterm birth status [20].

Reason for inclusion as an example. Dichotomisation of inflammation with no alternative grouping presented. Quantitative analysis also presented but not reported in abstract.

**Table 5:** Example of categorisation from the survey (3).

% optimal birth weight	Adjusted	
	Odds Ratio	95% CI
< 75	2.42	1.93,3.05
75-84	1.73	1.47,2.02
85-94	1.09	0.95,1.26
95-104	1 (referent)	
105-114	0.98	0.83,1.15
115-124	0.97	0.76,1.24
> 124	1.15	0.78,1.69

Risk of mild-moderate intellectual disability, by % optimal birth weight [21].

Reason for inclusion as an example. Seven groups in a large cohort, reference is middle group. Numbers in each group were not provided in the table.

Typically we noted that an alternative, coarser categorisation was used in the case of subgroup analyses, presumably to avoid unduly small numbers per category. For example, Cauley et al. [22] used three groups, rather than the five groups of the primary analysis, to examine the relationship between bone mineral density and vertebral fractures in several sub-groups of participants. Similarly, Fang et al. [23] used four groups, rather than the six groups of the primary analysis (Table 6), when assessing effect modification of the relationship between time since bereavement (due to death of a child) and amyotrophic lateral sclerosis (ALS) to "increase power in smaller groups".

**Table 6:** Example of categorisation from the survey (4).

	No. of cases	No. of controls	Odds Ratio	95% CI
No bereavement	2589	12722	Referent	
Time since bereavement (yrs)				
≤ 5	24	180	0.6	0.4, 1.0
6-10	18	116	0.8	0.5, 1.2
11-15	8	107	0.4	0.2, 0.8
16-20	11	80	0.7	0.4, 1.3
≥ 21	44	265	0.8	0.6, 1.1

Risk of amyotrophic lateral sclerosis (ALS) for bereaved parents by years since bereavement [23].

Reason for inclusion as an example. Example of a 'never' category, reference is 'never' category.

Overall, we note that there was usually little discussion of why categorisation was performed or justification for the number of categories chosen. Justification provided could be for testing of non-linearity [17,24] or to detect a threshold effect [25].

*Choice of category boundaries*. Boundaries for the categories were chosen using quantiles (n = 17, 34%), equally-spaced intervals (n = 9, 18%), external criteria (n = 6, 12%), or by other means (n = 17, 34%) (Table 2). One article (2%) did not provide details.

All 10 cohort studies which used the 'quantiles' approach based it on the risk factor distribution in the entire cohort whilst the two case-control studies which used quantiles (Chen et al. [26] used quintiles and Tworoger et al. [27] quartiles) based it on the distribution in the controls only. For skewed risk factor distributions, non-equal categories could be used. For example, in a cross-sectional study, Matsunga et al. [19] used the 30^th^, 60^th ^and 90^th ^percentiles of the distribution of the exposure in all subjects as the cut-offs to create 4 groups (Table 3). As noted above, the authors also provided results from a two-group categorisation which combined the lower three groups to compare to the upper 10^th ^of the distribution.

Equally-spaced boundaries, for example 5- or 10-year age bands, were used by 9 (18%) articles. Such equally-spaced boundaries were also used for variables expressed as percentages. For example, in assessing the relationship between percentage of optimal birth weight and intellectual disability, Leonard et al. [21] used groups of width 10% (Table 5).

An 'external criteria' approach to categorisation was classified as one which used well-recognised, published boundaries for the risk factor. Six articles (12%) used such external groupings. For example, Brunner Huber et al. [28] used WHO guidelines for body mass index (underweight: < 18.5; normal weight: 18.5 -24.9; overweight: 25-29.9; obese ≥ 30 kg/m^2^) to examine the effect of obesity on oral contraceptive failure.

Other approaches to categorisation were used by 17 (34%) articles. For example, in assessing the relationship between blood levels of vitamin D and fracture, Roddam et al. [29] categorised the exposure according to "proposed levels of vitamin D deficiency" whilst Park et al. [30] used "pre-defined categories of total calcium and dairy food intakes to maximise contrasts and ensure comparability with other studies" in assessing the effect of calcium on prostate cancer.

*"Zero/Never" categories*. Ten (20%) of the fifty categorisations described were of risk factors with a spike/clumping at the zero level of that risk factor or with a 'never' exposed category (Table 2). In the former, the spike at zero was used to form a 'zero' category, for example, 'pack-years of smoking' [31] and 'average weekly drinks' [32]. An example of the latter was a case-control study to assess the effect of time since bereavement due to loss of a child on the risk of ALS [23], where most subjects (94%) had not lost a child. Thus, the exposure 'years since bereavement' contained a 'never' category (Table 6).

*Presentation of categorical results*. The majority (n = 37, 74%) of the fifty articles with a categorical analysis presented the results in tables only (Table 2). Another 5 (10%) also included a figure with a table, whilst 5 (10%) used figures only. A few articles (n = 3) provided neither tables nor figures. Such articles referred to having used categorisation simply for the purposes of exploring the relationship between the exposure and outcome rather than to present the results, e.g. Inskip et al. [25].

An example of a figure which has been used to summarise results from both a continuous and categorical analysis is presented in Figure 1. With this figure, Rosenlund et al. [17] clearly convey the relationship between NO_2 _exposure and incidence of first coronary event.

### Estimation and Inference

*Estimation*. Twenty-nine articles presented a continuous analysis (8 continuous only and 21 with both categorical and continuous) (Table 7). Of these, 23 (79%) presented a point estimate per 1-unit increase of the exposure variable, one presented the exposure effect as the difference between the 90^th ^and 10^th ^percentile [33], while five articles provided no point estimate. Twenty-two articles also provided a confidence interval (CI) or standard error (SE) for the estimate.

**Table 7:** Estimation and statistical testing by analysis type^a^.

	Analysis type			
	**Continuous** **(n = 8)**	**Categorical** **(n = 29)**	**Both** **(n = 21)**	**Overall** **(n = 58)**
Type of estimate - n				
Continuous	7	0	16	23 (40%)
By group for all groups	0	4	6	10 (17%)
By group relative to ref group	0	26	12	38 (66%)
Other	1^b^	1^c^	3^d^	5 (9%)
				
Type of statistical test - n				
Continuous	8	0	19	27 (47%)
Score trend test	0	11	1	12 (21%)
Median/mean trend	0	7	1	8 (14%)
Pairwise	0	17	9	26 (45%)
Global	0	3	6	9 (16%)
Other	0	0	1^e^	1 (2%)
				

For the 58 articles with a continuous risk factor.

^a ^More than one estimate type and more than one statistical test is possible: 40 (69%) articles had one type of estimate (8 from 'continuous', 27 from 'categorical' and 5 from 'both') whilst 18 (31%) articles had two types of estimate (2 from 'categorical' and 16 from 'both'); 35 (60%) articles had one type of statistical test (8 from 'continuous', 20 from 'categorical' and 7 from 'both'); 21 (36%) articles had two types of statistical test (9 from 'categorical' and 12 from 'both') and 2 (3%) articles (from 'both') had three types of statistical test.

^b ^A continuous analysis estimate given as difference between 90^th ^and 10^th ^percentile.

^c ^Reference group is the background population overall i.e. standardised incidence.

^d ^One article gave hazard ratios per one category increase, another article compared 1^st ^and 4^th ^quartiles only, the final article reported the mean by categories.

^e ^A t-test comparing means in two outcome groups.

Fifty articles presented categorical analyses (29 categorical only and 21 both continuous and categorical). Of these, 10 (20%) presented an estimate by group for all groups usually (50%) accompanied by a CI or SE. Thirty-eight (76%) articles presented an estimate by group relative to a reference group, accompanied by a CI or SE in all but one case. Of those, most (n = 30, 79%) selected an extreme category (highest: n = 5, 13%; lowest: n = 25, 66%) as the reference group, whilst 21% (n = 8) chose a category in the middle of the distribution. When selecting the reference group of non-equally distributed categories, the largest group may be selected. For example, in categorising calcium intake to assess its effect on prostate cancer risk, Park et al. [32] selected the second of six predefined groups which, we note, is the largest group.

*Inference*. Most articles reported results in terms of statistical significance, either by formal hypothesis testing (i.e. by reporting p-values) or by an inferred hypothesis test via interpretation of confidence intervals (Table 7). Of the 29 articles with a continuous analysis, 27 performed statistical testing either formally by use of p-values (19 articles) or implicitly by interpreting confidence intervals (8 articles). Of the 50 articles with a categorical analysis, 20 used some kind of trend test: most commonly by assigning a score to each category (where a 1-unit increase in the score corresponded to moving to the next highest category in the order of categories). One alternative was to assign a category mean or median to each member of a category and then analyse that as a continuous variable in the appropriate statistical model, e.g. Park et al. [32]. Overall, global tests across all categories without use of their ordering were used in 8 articles, all using p-values. Pairwise tests comparing all groups to a reference group were used in 26 articles, 11 by p-values and 15 via interpretation of confidence intervals.

## Discussion

Understanding the relationship between a continuous exposure variable (risk factor) and a health outcome involves determining the direction, strength and shape of that relationship. Our survey demonstrates that categorisation is commonly used (86% of such articles surveyed), as was seen in an earlier survey (84% of such articles surveyed) [14]. With its breadth of information, the current survey has shown that there is great diversity in practice. The categorisation of continuous confounding variables and continuous outcome variables, although not addressed in this article, also plays an important role in the analysis of epidemiological data.

### Motivation for categorisation

Much research exists detailing the advantages and disadvantages of categorisation vs. analysing variables continuously (Appendix 2). From a statistical viewpoint, categorisation of a continuous variable can often result in a loss of statistical efficiency [4-6]. Some authors [10,11] advise against the practice of categorisation irrespective of the number of categories.

More practical considerations, however, may favour categorisation for ease of interpretation of parameter estimates, their presentation to less statistically-minded public health professionals, and may be motivated by use of clinically relevant 'cut points' if they exist. Categorisation is often used in conjunction with a continuous analysis, for example, to check for, or model, non-linear effects. Some authors [12,13] have advocated the use of alternative, more flexible modelling approaches using the continuous variable such as spline regression modelling and generalised additive models to model non-linear outcome-exposure relationships, which avoid the need to categorise. Such modelling approaches are more statistically complex which may limit their wide-spread use, and can pose difficulties of interpretation e.g. is statistical uncertainty adequately expressed, and does the model extrapolate beyond the observed range of the data.

The decision to categorise a continuous risk factor should be made in light of the various advantages and disadvantages and will differ for each specific situation. For example, in examining the relationship between biomarkers of inflammation and mortality using a continuous analysis only [16], the analyses may have benefitted from a categorical analysis: a hazard ratio per 1 unit increase in the biomarker is not as readily interpretable.

Despite recommendation 11 of the STROBE statement [15] which advises authors to explain how continuous variables were analysed and to describe how and why groupings were chosen, few authors in our survey described their reasons for categorising a continuous variable. Ideally, choice and rationale for categorisation should be made prior to data analysis and documented in the publication.

### Specific Choice of Categorisation

When categorisation of a continuous risk factor is performed, decisions on the nature of the categorisation are needed i.e. the number of categories and the choice of cut-points. These may differ depending on the reason for categorisation and the size of the study. For example, a larger number of groups may be used when the study is large or when the purpose of categorisation is to check for non-linear effects. When the purpose is to assess effect modification (subgroup analyses) lack of statistical power may necessitate fewer groups.

There is much theoretical work on the nature of categorisation [4-6]. Equally-sized groups (such as using quartiles) have the merit of objective simplicity but are not the most statistically efficient choice. For instance, with Normal data it is more efficient to have larger groups in the centre of the distribution making the extreme groups smaller and hence even more extreme. Statistical efficiency increases with the number of groups [4,6], i.e. the more crude the grouping (e.g. 2 groups), the greater the loss of statistical power. Statistical efficiency is usually greatest using a continuous analysis, provided the model fit (e.g. linearity assumption) is good.

In selecting the category boundaries it is necessary to determine how many categories will be formed as sufficient individuals/events are needed in each group. Sensibly, only studies with large sample size or a strong exposure-outcome relationship would be able to support a large number of categories. If 'natural' or clinically important cut-points are relevant for the exposure variable then it is still important to verify that sufficient information is present in each of the categories for a robust analysis. If not, merging of some of the categories may be required.

Dichotomisation of the exposure variable is strongly advised against. In terms of statistical power, it is equivalent to discarding a third of the data [1,2] and makes it impossible to detect non-linearity in the exposure-outcome relationship. In our survey, no article solely presented results from dichotomisation, but three articles did place more emphasis on dichotomised results.

Multiple alternative categorisations should be undertaken with care and interpreted cautiously, as deliberate or subconscious data dredging could lead to a choice of grouping that accentuates an association thus increasing the risk of a false positive finding, and/or an exaggerated estimate of the exposure/outcome relationship. However, additional investigation of effect modification (subgroup analyses) may necessitate a secondary categorisation with fewer groups.

Inevitably readers can only assess categorisations reported in publications: other categorisation choices may have been analysed but not included. As a result, our survey of current practice is limited to what is published with no awareness of authors' selections in what they chose to report so that a full critical evaluation of those choices is not possible here. Authors usually did not explicitly report the reasons for the number of categories and choice of category boundaries, or specify whether these were chosen prior to analysis and whether they were the only categories explored.

### Estimation and Inference

Practice varies as to which contrasts are best used for estimation and for inference in a categorical analysis. Pairwise comparisons relative to a reference group was the most commonly reported approach in our survey, and they are easy to interpret. For such estimates, the highest or lowest category is usually (79%) chosen as the reference group possibly for ease of interpretation. However, if the largest group is not an extreme category, that may be a better choice of reference category for both statistical efficiency and practical reasons. Such multiple pairwise tests increase the chance of false positive results. Hence, a global test of the relationship between the categorised risk factor and the outcome is desirable. If the relationship between the risk factor and outcome appears to be monotonic, a trend test will be substantially more powerful and insightful than an unordered global test across multiple groups. It may also be helpful to report the estimate from a continuous analysis. All estimates, whether pairwise or otherwise, should be accompanied by either their standard error or 95% CI as an indication of statistical uncertainty.

### Presentation and reporting of results

Most articles in our survey reported the results of their categorical analysis in table form only (74%); whereas only 20% (10 articles) used figures and 6% (3 articles) used neither. We would encourage a greater use of figures as a valuable way of visually conveying information across categories to the reader as demonstrated by the example shown in Figure 1 [17]. Ideally, the number of patients and, if relevant, the number of events and estimates in each category should also be tabulated on the figure e.g. as numbers under the x-axis. If multiple exposure variables are of interest, space constraints may prevent all results being reported as figures.

Care should be taken in the choice of results to report in the article's abstract. These should accurately reflect the analysis as a whole and avoid only reporting statistically significant results, particularly if multiple categorisations of the same risk factor were undertaken with differing conclusions

### Recommendations

To date, there exist few guidelines on the practice of categorisation in epidemiological publications [9]. Our survey has provided a number of lessons. It is useful to combine these with ideas from experience and from the broader literature into a series of recommendations which may complement the STROBE guidelines [15]. In practice it is not sensible to propose a "one-size-fits-all" approach but some guidance as to what to consider when undertaking and reporting categorisation would be helpful. The overarching goal of such guidance is to encourage authors to report the number of selected categories, the rationale for the selection of category boundaries, and whether these were determined entirely before the data were analysed or guided, at least in part, by the data analysis. Accordingly, we offer the following pointers for authors:

1. Be aware of the advantages and disadvantages of categorisation (Appendix 2).

2. Define (as far as possible) the chosen categories prior to analysis but be careful to not miss interesting hypothesis generating opportunities, especially in large studies.

3. Consider the distribution of the data when choosing categories: for skewed exposure distributions, consider cut-points which sensibly capture the tail of the distribution; for more symmetric distributions, refer to the theoretical literature [5,6] in considering whether to deviate from grouping into equal sized groups.

4. Report clearly the reasons for categorisation and the specific chosen boundaries.

5. Take care when choosing the number of categories, bearing in mind the extent of data available i.e. large studies may permit a large number of categories.

6. It is best to avoid dichotomisation.

7. Be wary of injudicious use of multiple alternative categorisations, especially if done to artificially accentuate associations.

8. Consider use of figures to more clearly visualise the pattern of outcomes across categories.

9. Provide numbers of participants and events by category in appropriate tables and figures.

10. Consider use of an appropriate trend test across groups.

11. Provide confidence intervals for point estimates within each group, or appropriate estimates and confidence intervals of pairwise between-group differences.

12. Take care in choosing the appropriate estimates (and significance tests) for association, especially regarding the choice of reference group for pairwise group comparisons or the wisdom of a more global monotonic trend across groups.

13. Consider presenting results from continuous analysis including statistical modelling to account for non-linear associations (e.g. spline modelling, generalised additive models), though beware of potential model instabilities and over interpretation.

## Conclusions

There exists a healthy debate concerning the advantages and disadvantages of categorisation. Some are of the opinion that categorisation should not be used even going so far as to state that "categorisation of continuous data is not necessary, and indeed is not a natural way of analyzing continuous data for most statisticians" [pg566, 10]. We venture the alternative view that the categorisation of continuous risk factors has, and will likely continue to play, an important role in the analysis of epidemiological data.

In this article we have focused on the diversity of current practice in the use of categorisation in epidemiological studies. We hope that our survey, critical appraisal and consequent recommendations regarding how categorisation (grouping) is, and should be, presented will be of value to future authors and journals in enhancing the quality of epidemiological publications.

## Competing interests

The authors declare that they have no competing interests.

## Authors' contributions

The survey was initiated by SJP. All authors contributed equally to the design of the study. ELT and JED independently surveyed the literature with support from SJP. All authors contributed to the writing and editing of the manuscript, and have approved the final version.

## Appendix 1

### Survey information collected from eligible epidemiological publications

Type of study design (case-control, cohort, cross-sectional)

Main outcome

• Type (binary, time-to-event, ordered categorical, unordered categorical, continuous and other)

Main continuous risk factor

• *Characteristics:*

- Primary outcome measure (e.g. odds ratio, rate ratio)

- Type of analysis i.e. treated as a continuous variable only, as a categorical variable only or both; if 'both', was emphasis on continuous or categorised form

- Number of other continuous risk factors categorised in the same manner

• *Nature of the categorisation:*

- Number of categories

- Criteria used to select boundaries of categories (i.e. quantiles; equally-spaced groups; external criteria where an explicit reference to well-recognised boundaries was provided; other)

- Number of alternative categorisations

- Inclusion of a "zero/never" category

• *Details of the analysis:*

- Estimation: type of estimate (continuous; by group for all groups; by group relative to reference group; other) and reporting of confidence intervals

- Statistical testing: type of test (continuous analysis; trend test; pairwise comparisons; global test; other) and reporting of p-values

• *Presentation of categorical results:*

Tables, figures or both

## Appendix 2

### Advantages and disadvantages of categorisation of continuous risk factors

#### Advantages of categorisation

• Presentation of results may be simpler to understand by non-statisticians. For instance, some people may find risks presented relative to a reference group easier to interpret than regression coefficients or correlation coefficients.

• Results may relate more directly to individuals and thus be more readily interpretable. For example, a relative risk for a high category versus a low category subject could be obtained.

• There may be a natural or conventional form of categories that should be used. For example, SBP < 140, 140-159, ≥160 mmHg.

• Categorisation may remove the need for any parametric assumptions regarding the shape (e.g. linearity) of the outcome/exposure relationship.

• A 'never' or 'zero' exposed category can be easily incorporated into a categorical analysis e.g. 'no bereavement' for the exposure 'years since bereavement'.

#### Disadvantages of categorisation

• No single "right answer", as different choices of categories may lead to somewhat different findings, and sometimes conclusions may actually differ.

• No agreed objective criteria on the number of groups to choose or the boundaries (cut-off points) for grouping.

• No agreement on which contrasts to use for inference, e.g. whether to use trend test or pairwise comparisons.

• No agreement whether to use an extreme (i.e. lowest or highest) or middle (most common) group as the reference.

• Deliberate or subconscious data dredging could lead to a choice of grouping that accentuates an association thus increasing the risk of a false positive finding, and/or an exaggerated estimate of the exposure/outcome relationship.

• Statistical power/efficiency is lost compared to a continuous variable in regression.

• Continuous modelling can potentially give greater insight.

## Supplementary Material

Additional file 1 **Proforma for survey of categorisation in observational epidemiology articles**. Click here for file

Additional file 2 **References for the 58 articles included in the survey (in journal and date/page number order)**. Click here for file
