# Supplementary material for: Categorisation of continuous risk factors in epidemiological publications: a survey of current practice
Source: Epidemiol Perspect Innov. 2010 Oct 15;7:9. doi: 10.1186/1742-5573-7-9 (PMC2972292; doi:10.1186/1742-5573-7-9)
Supplement: Additional file 1 — Proforma for survey of categorisation in observational epidemiology articles. [file 1742-5573-7-9-S1.doc]

**Additional file 1 - Proforma for survey of categorisation in observational epidemiology articles**
