# Supplementary material for: Categorisation of continuous risk factors in epidemiological publications: a survey of current practice
Source: Epidemiol Perspect Innov. 2010 Oct 15;7:9. doi: 10.1186/1742-5573-7-9 (PMC2972292; doi:10.1186/1742-5573-7-9)
Supplement: Additional file 2 — References for the 58 articles included in the survey (in journal and date/page number order). [file 1742-5573-7-9-S2.doc]

### Additional file 2 – References for the 58 articles included in the survey (in journal and date / page number order)

1. Edwards CG, Schwartzbaum JA, Nise G, Forssen UM, Ahlbom A, Lonn S, Feychting M. Occupational noise exposure and risk of acoustic neuroma. Am. J. Epidemiol. 2007; 166: 1252-1258.
2. Park S-Y, Murphy SP, Wilkens LR, Stram DO, Henderson BE, Kolonel LN. Calcium, vitamin D, and dairy product intake and prostate cancer risk: the multiethnic cohort study. Am. J. Epidemiol. 2007; 166: 1259-1269.
3. Park Y, Mitrou PN, Kipnis V, Hollenbeck A, Schatzkin A, Leitzmann MF. Calcium, dairy foods, and risk of incident and fatal prostate cancer: the NIH-AARP diet and health study. Am. J. Epidemiol. 2007; 166: 1270-1279.
4. Kifley A, Liew G, Wang JJ, Kaushik S, Smith W, Wong TY, Mitchell P. Long-term effects of smoking on retinal microvascular caliber. Am. J. Epidemiol. 2007; 166: 1288-1297.
5. Stevens J, Murray DM, Baggett CD, Elder JP, Lohman TG, Lytle LA, Pate RR, Pratt CA, Treuth MS, Webber LS, Young DR. Objectively assessed associations between physical activity and body composition in middle-school girls: the Trial of Activity for Adolescent Girls. Am. J. Epidemiol. 2007; 166: 1298-1305.
6. Brunner Huber LR, Toth JL. Obesity and oral contraceptive failure: findings from the 2002 national survey of family growth. Am. J. Epidemiol. 2007; 166: 1306-1311.
7. Catov JM, Bodnar LM, Ness RB, Barron SJ, Roberts JM. Inflammation and dyslipidemia related to risk of spontaneous preterm birth. Am. J. Epidemiol. 2007; 166: 1312-1319.
8. Roddam AW, Neale R, Appleby P, Allen NE, Tipper S, Key TJ. Association between plasma 25-hydroxyvitamin D levels and fracture risk: the EPIC-Oxford study. Am. J. Epidemiol. 2007; 166: 1327-1336.
9. Romundstad PR, Davey Smith G, Nilsen TIL, Vatten LJ. Associations of prepregnancy cardiovascular risk factors with the offspring's birth weight. Am. J. Epidemiol. 2007; 166: 1359-1364.
10. Jacobsen BK, Heuch I, Kvale G. Association of low age at menarche with increased all-cause mortality: a 37-year follow-up of 61,319 Norwegian women. Am. J. Epidemiol. 2007; 166: 1431-1437.
11. Varraso R, Jiang R, Barr RG, Willett WC, Camargo CA Jr. Prospective study of cured meats consumption and risk of chronic obstructive pulmonary disease in men. Am. J. Epidemiol. 2007; 166: 1438-1445.
12. Gao X, Chen H, Schwarzschild MA, Glasser DB, Logroscino G, Rimm EB, Ascherio A. Erectile function and risk of Parkinson's disease. Am. J. Epidemiol. 2007; 166: 1446-1450.
13. Vercambre MN, Fournier A, Boutron-Ruault M-C, Clavel-Chapelon F, Ringa V, Berr C. Differential dietary nutrient Intake according to hormone replacement therapy use: an underestimated confounding factor in epidemiologic studies? Am. J. Epidemiol. 2007; 166: 1451-1460.
14. Mukamal KJ, Kennedy M, Cushman M, Kuller LH, Newman AB, Polak J, Criqui MH, Siscovick DS. Alcohol consumption and lower extremity arterial disease among older adults: the cardiovascular health study. Am. J. Epidemiol. 2008; 167: 34-41.
15. Ohira T, Hozawa A, Iribarren C, Daviglus ML, Matthews KA, Gross MD, Jacobs DR Jr. Longitudinal association of serum carotenoids and tocopherols with hostility: the CARDIA study. Am. J. Epidemiol. 2008; 167: 42-50.
16. Wong TY, Cheung N, Islam FMA, Klein R, Criqui MH, Cotch MF, Carr JJ, Klein BEK, Richey Sharrett A. Relation of retinopathy to coronary artery calcification: the multi-ethnic study of atherosclerosis. Am. J. Epidemiol. 2008; 167: 51-58.
17. Takachi R, Inoue M, Ishihara J, Kurahashi N, Iwasaki M, Sasazuki S, Iso H, Tsubono Y, Tsugane S, for the JPHC Study Group. Fruit and vegetable intake and risk of total cancer and cardiovascular disease: Japan public health center-based prospective study. Am. J. Epidemiol. 2008; 167: 59-70.
18. Thurston RC, Sowers MFR, Chang Y, Sternfeld B, Gold EB, Johnston JM, Matthews KA. Adiposity and reporting of vasomotor symptoms among midlife women: the study of women's health across the nation. Am. J. Epidemiol. 2008; 167: 78-85.
19. Chen H, O'Reilly EJ, Schwarzschild MA, Ascherio A. Peripheral inflammatory biomarkers and risk of Parkinson's disease. Am. J. Epidemiol. 2008; 167: 90-95.
20. Leonard H, Nassar N, Bourke J, Blair E, Mulroy S, de Klerk N, Bower C. Relation between intrauterine growth and subsequent intellectual disability in a ten-year population cohort of children in Western Australia. Am. J. Epidemiol. 2008; 167: 103-111.
21. Beam Dowd J, Haan MN, Blythe L, Moore K, Aiello AE. Socioeconomic gradients in immune response to latent infection. Am. J. Epidemiol. 2008; 167: 112-120.
22. Yang J, Carmichael SL, Canfield M, Song J, Shaw GM, National Birth Defects Prevention Study. Socioeconomic status in relation to selected birth defects in a large multicentered US case-control study. Am. J. Epidemiol. 2008; 167: 145-154.
23. Inskip HM, Dunn N, Godfrey KM, Cooper C, Kendrick T, Southampton Women's Survey Study Group. Is birth weight associated with risk of depressive symptoms in young women? Evidence from the Southampton women's survey. Am. J. Epidemiol. 2008; 167: 164-168.
24. Heikura U, Taanila A, Hartikainen A-L, Olsen P, Linna S-L, von Wendt L, Jarvelin M-R. Variations in prenatal sociodemographic factors associated with intellectual disability: a study of the 20-year interval between two birth cohorts in northern Finland. Am. J. Epidemiol. 2008; 167: 169-177.
25. Gunderson EP, Rifas-Shiman SL, Oken E, Rich-Edwards JW, Kleinman KP, Taveras EM, Gillman MW. Association of fewer hours of sleep at 6 months postpartum with substantial weight retention at 1 year postpartum. Am. J. Epidemiol. 2008; 167: 178-187.
26. Purslow LR, Sandhu MS, Forouhi N, Young EH, Luben RN, Welch AA, Khaw K-T, Bingham SA, Wareham NJ. Energy intake at breakfast and weight change: prospective study of 6,764 middle-aged men and women. Am. J. Epidemiol. 2008; 167: 188-192.
27. Bjerkeset O, Romundstad P, Evans J, Gunnell D. Association of adult body mass index and height with anxiety, depression, and suicide in the general gopulation: the HUNT study. Am. J. Epidemiol. 2008; 167: 193-202.
28. Fang F, Ye W, Fall K, Lekander M, Wigzell H, Sparen P, Adami H-O, Valdimarsdóttir U. Loss of a child and the risk of amyotrophic lateral sclerosis. Am. J. Epidemiol. 2008; 167: 203-210.
29. Tworoger SS, Lee I-M, Buring JE, Hankinson SE. Plasma androgen concentrations and risk of incident ovarian cancer. Am. J. Epidemiol. 2008; 167: 211-218.
30. Brindel P, Doyon F, Rachedi F, J-L Boissin, Sebbag J, Shan L, Chungue V, Sun LYK, Bost-Bezeaud F, Petitdidier P, Paoaafaite J, Teuri J, de Vathaire F. Menstrual and reproductive factors in the risk of differentiated thyroid carcinoma in native women in French Polynesia: a population-based case-control study. Am. J. Epidemiol. 2008; 167: 219-229.
31. Li CI, Malone KE, Daling JR, Potter JD, Bernstein L, Marchbanks PA, Strom BL, Simon MS, Press MF, Ursin G, Burkman RT, Folger SG, Norman S, McDonald JA, Spirtas R. Timing of menarche and first full-term birth in relation to breast cancer risk. Am. J. Epidemiol. 2008; 167: 230-239.
32. Knudtson MD, Klein R, Lee KE, Reinke JO, Danforth LG, Wealti AM, Moore E, Klein BEK. A longitudinal study of nonvitamin, nonmineral supplement use: prevalence, associations, and survival in an aging population. Ann. Epidemiol. 2007; 17: 933-939.
33. Hartz AJ, Daly JM, Kohatsu ND, Stromquist AM, Jogerst GJ, Kukoyi OA. Risk factors for insomnia in a rural population. Ann. Epidemiol. 2007; 17: 940-947.
34. Askarian M, Shaghaghian S, McLaws M-L. Needlestick injuries among nurses of Fars Province, Iran. Ann. Epidemiol. 2007; 17: 988-992.
35. Tanaka K, Miyake Y, Arakawa M, Sasaki S, Ohya Y. Prevalence of asthma and wheeze in relation to passive smoking in Japanese children. Ann. Epidemiol. 2007; 17: 1004-1010.
36. Tsai SP, Ahmed FS, Wendt JK, Bhojani F, Donnelly RP. The impact of obesity on illness absence and productivity in an industrial population of petrochemical workers. Ann. Epidemiol. 2008; 18: 8-14.
37. Barrett RE, Cho YI, Weaver KE, Ryu K, Campbell RT, Dolecek TA, Warnecke, RB. Neighbourhood change and distant metastasis at diagnosis of breast cancer. Ann. Epidemiol. 2008; 18: 43-47.
38. Brotman RM, Klebanoff MA, Nansel T, Zhang J, Schwebke JR, Yu KF, Zenilman JM, Andrews WW. Why do women douche? A longitudinal study with two analytical approaches. Ann. Epidemiol. 2008; 18: 65-73.
39. Gonzalez A, Peters U, Lampe JW, Satia JA, White E. Correlates of toenail zinc in a free-living US population. Ann. Epidemiol. 2008; 18: 74-77.
40. Matsunga I, Miyake Y, Yoshida T, Miyamoto S, Ohya Y, Sasaki S, Tanaka K, Oda H, Ishiko O, Hirota Y, and The Osaka Maternal and Child Health Study Group. Ambient formaldehyde levels and allergic disorders among Japanese pregnant women: baseline data from the Osaka Maternal and Child Health Study. Ann. Epidemiol. 2008; 18: 78-84.
41. Galea S, Ahern J, Tracey M, Hubbard, A, Cerda M, Goldmann E, Vlahov D. Longitudinal determinants of posttraumatic stress in a population-based cohort study. Epidemiology. 2008; 19: 47-54.
42. Savitz D, Chan RL , Herring AH, Howards PP, Hartmann KE. Caffeine and miscarriage risk. Epidemiology. 2008; 19: 55-62.
43. Vestergarrd H, Westergaard T, Wohlfahrt J, Pipper C, Melbye M. Association between intussusception and tonsil disease in childhood. Epidemiology. 2008; 19: 71-74.
44. Rosenlund M, Picciotto S, Forastiere F, Stafoggia M, Perucci CA. Traffic-related air pollution in relation to incidence and prognosis of coronary heart disease. Epidemiology. 2008; 19: 121-128.
45. Oftedal B, Brunekreef B, Nystad W, Madsen C, Walker S-E, Nafstad P. Residential outdoor air polllution and lung function in schoolchildren. Epidemiology. 2008; 19: 129-137.
46. Auchincloss AH, Diez R, Ana V, Brown DG, Erdmann CA, Bertoni AG. Neighborhood resources for physical activity and healthy foods and their association with insulin resistance. Epidemiology. 2008; 19: 146-157.
47. Ford AC, Forman D, Bailey AG, Goodman KJ, Axon ATR, Moayyedi P. Effect of sibling number in the household and birth order on prevalence of Helicobacter pylori: a cross-sectional study. Int. J. Epidemiol. 2007; 36: 1327-1333.
48. Reeves GK, Pirie K, Beral V, Green J, Spencer E, Bull D. Cancer incidence and mortality in relation to body mass index in the Million Women Study. Br. Med. J. 2007; 355: 1134.
49. Sui X, LaMonte MJ, Laditka JN, Hardin JW, Chase N, Hooker SP, Blair SN. Cardiorespiratory fitness and adiposity as mortality predictors in older adults. J. Am. Med. Assoc. 2007; 298: 2507-2516.
50. Cauley JA, Hochberg MC, Lui L-Y, Palermo L, Ensrud KE, Hillier TA, Nevitt MC. Long-term risk of incident vertebral fractures. J. Am. Med. Assoc. 2007; 298: 2761-2767.
51. Begg CB, Haile RW, Borg A, Malone KE, Concannon P, Thomas DC, Langholz B, Bernstein L, Olsen JH, Lynch CF, Anton-Cluver H, Capunu M, Liang X, Hummer AJ, Sima C, Bernstein JL. Variation of breast cancer risk among BRCA1/2 carriers. AJ. Am. Med. Assoc. 2008; 299: 194-201.
52. Bartali B, Frongilo EA, Guralnik JM, Stipanuk MH, Allore HG, Cherubini A, Bandinelli S, Ferrucci L, Gill TM. Serum micronutrient concentrations and decline in physical function among older persons. J. Am. Med. Assoc. 2008; 299: 308-315.
53. Baker JL, Olsen LW, Sorensen TIA. Childhood body-mass index and the risk of coronary heart disease in adulthood. N. Engl. J. Med. 2007; 357: 2329-2337.
54. Downs SH, Schindler C, Liu L-J S, Keidel D, Bayer-Oglesby L, Brutsche MH, Gerbase MW, Keller R, Kunzli N, Leuenberger P, Probst-Hensch NM, Tschopp J-M, Zellweger J-P, Rochat T, Schwartz J, Ackermann-Liebrich U, and the SAPALDIA Team. Reduced exposure to PM10 and attenuated age-related decline in lung function. N. Engl. J. Med. 2007; 357: 2338-2347.
55. Chan PS, Krumholz HM, Nichol G, Nallamothu BK, and the American Heart Association National Registry of Cardiopulmonary Resuscitation Investigators. Delayed time to defibrillation after in-hosptial cardiac arrest. N. Engl. J. Med. 2008; 358: 9-17.
56. Semba RD, de Pee S, Sun K, Sari M, Akhter N, Bloem MW. Effect of parental formal education on risk of child stunding in Indonesia and Bangladesh: a cross-sectional study. Lancet. 2008; 371: 322-328.
57. Lauer MS, Pothier CE, Majid DJ, Smith SS, Kattan MW. An externally validated model for predicting long-term survival after exercise treatmill testing in patients with suspected coronary artery disease and a normal electrocardiogram. Ann. Intern. Med. 2007; 147: 821-828.
58. Vidula H, Tian L, Liu K, Criqui MH, Ferrucci L, Pearce WH, Greenland P, Green D, Tan J, Garside DB, Guralnik J, Ridker PM, Rifai N, McDermott MM. Biomarkers of inflammation and thrombosis as predictors of near-term mortality in patients with peripheral arterial disease: a cohort study. Ann. Intern. Med. 2008; 148: 85-93.
